# Three-Dimensional Graphene-Based Composite Hydrogel Materials for Flexible Supercapacitor Electrodes

**DOI:** 10.3389/fchem.2019.00660

**Published:** 2019-10-01

**Authors:** Enping Lai, Xinxia Yue, Wan'e Ning, Jiwei Huang, Xinlong Ling, Haitao Lin

**Affiliations:** Guangxi Key Laboratory of Green Processing of Sugar Resources, College of Biological and Chemical Engineering, Guangxi University of Science and Technology, Liuzhou, China

**Keywords:** flexible supercapacitor, electrode materials, grahpene-based hydrogel, three-dimensional architecture, composite materials

## Abstract

Three-dimensional (3D) graphene-based hydrogels have attracted great interest for applying in supercapcacitors electrodes, owing to their intriguing properties that combine the structural interconnectivities and the outstanding properties of graphene. However, the pristine graphene hydrogel can not satisfy the high-performance demands, especial in high specific capacitance. Consequently, novel graphene-based composite hydrogels with increased electrochemical properties have been developed. In this mini review, a brief summary of recent progress in the research of the three-dimensional graphene-based composite hydrogel for flexible supercapacitors electrodes materials is presented. The latest progress in the graphene-based composite hydrogel consisting of graphene/metal, graphene/polymer, and atoms doped graphene is discussed. Furthermore, future perspectives and challenges in graphene-based composite hydrogel for supercapacitor electrodes are also expressed.

## Introduction

In recent years, supercapacitors have been attracted intensive investigation for sustainable energy application, because of their advantages in excellent power density and high charge/discharge rates (Li et al., 2018). With the rapid growth of wearable electronics, flexible supercapacitors that can work under consecutive bending or stretching are urgently needed. However, it remains a great challenge to obtain supercapacitors with excellent electrochemical performance and good flexibility. Since the supercapacitors mainly consist of two electrodes and a separated membrane, the electrodes are often considered to be a key factor (Xu et al., 2018). Thus, it is critically important to develop innovative materials for flexible supercapacitor electrodes.

As a unique two-dimensional (2D) carbon material, graphene has gained tremendous attention in various application aspects due to its fast-charged carrier mobility, excellent conductivity and largely tunable surface area (Sattar, 2019). Based on the extraordinary physico-chemical properties, graphene and its functionalized derivatives (graphene oxide, GO) can be applied in supercapacitors as the electrode materials. Previous studies have demonstrated that the supercapacitors based on graphene can possess excellent specific capacitance (Horn et al., 2019). Unfortunately, the restacking or irreversible agglomeration of graphene sheets can suppress the high conductivity and decrease accessible surface area, which limit the improvement of capacitive performance. To tackle these challenges, three-dimensional (3D) graphene-based architectures including foam, hydrogels, sponges are developed.

Among various 3D macroscopic structures, graphene hydrogels consist of interconnected porous networks with large specific surface areas have received particular attention (Lu et al., 2017). These hydrogels provide multidimensional ion/electron transport pathways with the intrinsic properties of graphene, which makes them promising candidates for supercapacitor electrodes. Great achievements of supercapacitors based on graphene hydrogels have been obtained, while the pristine graphene hydrogel cannot meet the requirements in practical application (Ma et al., 2018). Functional materials or dopants such as metal oxides or hydroxide, conducting polymers, and so on have been introduced to the graphene hydrogels to further improve the electrochemical performance. In this mini-review, a brief retrospect on graphene-based composite hydrogel materials for flexible supercapacitor electrodes will be provided.

## Graphene-Based Composite Hydrogel Electrode Materials

### Graphene-Metal Composite Hydrogels Electrode Materials

Usually, metal oxides or hydroxides exhibit high pseudo-capacitance mainly due to their faradic reaction beyond formation of electrical double-layers in the charge-discharge processes. Thus, metal oxides or hydroxides including MnO_2_, Ni(OH)_2_, and etc. have been widely incorporated into graphene to form composite materials to obtain enhanced performance.

Researches have been focused on coupling MnO_2_ with other materials (Xu et al., 2019) for supercapacitor electrodes, and different structures of MnO_2_ have also been incorporated in graphene hydrogel. Zhang et al. (2016) prepared micro-nanostructured pompon-like MnO_2_/graphene hydrogel composites, and the hydrogel with a MnO_2_ content of 50% displayed a good capacitive behavior (445.7 F/g at 0.5 A/g). Tran et al. (2017) synthesized graphene/α-MnO_2_ nanowire hydrogel with a high specific capacitance (397 F/g) at 1.0 A/g. Meng et al. (2018) designed a glucose and ammonia reduction system to synthesize δ-MnO_2_/graphene hydrogel with a capacity of 200.6 F/g.

As a transition metal hydroxide, Ni(OH)_2_ have been commonly used. In view of the structure, the Ni(OH)_2_ nanoplate (Mao et al., 2016) and nanoflower structure (Wang et al., 2016) have been designed to form 3D graphene-based frameworks, and the capacitance can be achieve to 782 F/g at 0.2 A/g and 1,632 F/g at 1 A/g, respectively. Recently, Li et al. (2019) coupled Ni(OH)_2_ nanosheets with nitrogen-enriched graphene hydrogel, which featured a specific capacitance of 896 F/g at 0.5 A/g.

Besides, in consideration of the advantage of Co and Ni, the strategy of combining Ni with Co was developed. Hwang et al. (2018) embedded Ni-Co hydroxide nanoneedles in graphene hydrogel, and the nanocomposite exhibited a specific capacitance of 544 C/g at 2 A/g. Tiruneh et al. (2018) designed a binder-free hybrid graphene hydrogel with nickel cobalt sulfide embedded, which can exhibit a capacity of about 1,000 F/g at 0.75 A/g with outstanding stability.

In addition, as one of the most promising nanomaterials, TiO_2_ has also been focused. Liu et al. (2017) obtained rice-like TiO_2_/graphene composite hydrogel, and the interaction between TiO_2_ nanoparticles and graphene hydrogel can endow the composite hydrogel with superior physicochemical properties, resulting in an excellent capacity of 372.3 F/g at 0.2 A/g. In addition, to realize synergic effect of organic and inorganic materials, Zhang et al. (2018b) synthesized RuO_2_/graphene hydrogel, and then adsorbed 1,4-naphthoquinone (NQ) molecules onto the hydrogel to form an hybrid graphene hydrogel, and the hydrogel containing ~14.6% RuO_2_ also can show a superior specific capacitance of 450.8 F/g.

### Graphene-Polymer Composite Hydrogels Electrode Materials

Conductive polymers with the reverse doping-dedoping behavior have been extensively incorporated into 3D graphene hydrogels to fabricate high-performance electrodes. One of the successful conducting polymers is polyaniline (PANI), which has good environmental stability and high pseudocapcacitance. Various types of PANI including nanorods and nanowires have been used to form graphene/PANI composite hydrogel, and these hydrogels can show improved specific capacitance (Chen et al., 2017; Xu et al., 2017). To retain the essential features of the native hydrogel, the thin PANI layer wrapped on the graphene hydrogel by Gao et al. (2016) using an electrodeposition method, and the hydrogel displayed a good specific capacitance (710 F/g at 2 A/g). In addition, the hydrogel structure and the preparation temperature are also important. Wu et al. (2016) found that the phase-separated structure can produce much channels for electrolyte diffusion in PANI/graphene hydrogel, leading to a specific capacitance as high as 783 F/g at 27.3 A/g. Zou et al. (2018b) used *m*-phenylenediamine (*m*PD) to preserve the conjugated structure of PANI, and the composite hydrogel showed an improved capacity of 514.3 F/g. They also developed double-crosslinked network functionalized graphene/PANI hydrogel with high specific capacitance and mechanical strength (Zou et al., 2018a). For the preparation temperature, Ates et al. (2018) showed that the high capacitance can be kept at ~99% of its pristine value for the hydrogel prepared at 25°C, in comparison of that at 0°C.

Due to the ultrahigh theoretical capacitance and mechanical flexibility, polypyrrole (PPy) has also attracted considerable attention. Wu and Lian (2017) synthesized graphene/PPy hydrogel with a specific capacitance of 363 F/cm^3^ at 1.0 mA/cm^3^ using hydroquinone as a functionalized molecule. Since the ion transportation may be restricted by the compact morphology of PPy/graphene composite, the strategy of PPy wrapped graphene was developed. Pattananuwat and Aht-ong (2017) controlled nanoporous structure for PPy coated on graphene hydrogel surface with aiding of the surfactant, and a high specific capacitance (640.8 F/g at 1 A/g) can be achieved. They also used poly(3,4-ethylenedioxythiophene) (PEDOT) with PPy to form the synergistic effect, and the resultant hydrogel exhibited a specific capcacitance of 342 F/g at 0.5 A/g (Pattananuwat and Aht-ong, 2016). Recently, a hybrid PPy/rGO hydrogel *in-situ* electropolymerization preparation of PPy on the outside layer of graphene was reported by Zhang et al. (2019), and the specific capacitance is 340 F/g at 1 A/g.

Except for PANI and PPy, biopolymer such as lignosulfonate that a derivate of lignin can be regarded as a candidate for electrode material due to its electroactive components. Xiong et al. (2016) prepared lignosulfonate/graphene hydrogel with a maximum capacity of 549.5 F/g at 1 A/g. Li et al. (2017a) also reported lignosulfonate functionalized graphene hydrogels, which can present a specific capacitance of 432 F/g. These renewable composite hydrogels exhibit great potential in supercapacitor as electrodes materials.

### Doped Graphene Composite Hydrogels Electrode Materials

As known, chemical doping is an effective strategy to modify the intrinsic properties of the materials. For graphene materials, nitrogen doping has been found to be an important method to provide graphene composite with high capacitance (Jin et al., 2019). For example, Liao et al. (2016) used urea and a small amount of ammonia to prepare N-doped graphene hydrogel, and the different N-types in graphene can resulted in an excellent specific capacitance (387.2 F/g at 1 A/g). Except for urea and ammonia, other materials containing N also have been used. Jiang et al. (2016) designed N-doped graphene hydrogel with a good specific capacitance (167.7 F/g at 1 A/g) by using carbohydrazide. Liu et al. (2016) used ammonium bicarbonate to prepared porous N-doped graphene hydrogel, and the hydrogel with high N content (10.8 at%) showed a high specific capacitance of 194.4 F/g. Gao et al. (2019) synthetized nitrogen-doped graphene-based hydrogels with concentrated sulfuric acid and o-phenylenediamine (oPDA), and the optimal hydrogel showed specific capacitance about 519.8 F/g.

Compared to single-atom doping, multiple doping can exhibit a synergetic effect and further improve the capacitive behavior of the materials. Typically, nitrogen and sulfur can be doped into graphene concurrently. After co-doping, the pseudo capacitance of the graphene will be increase, because of the redox faradic reactions existed at nitrogen-containing groups and sulf-containing species. Tran et al. (2016) outlined that N and S co-doped graphene hydrogel with holy defect can show a wonderful specific capacitance of 538 F/g at 0.5 mV/s, and the electrochemical property of the hydrogel can be modulated by the level of N and S doping. Li et al. (2017b) synthesized N/S co-doped graphene hydrogels with hierarchical pores, which can demonstrate a very good specific capacitance (251 F/g at 0.5 A/g) surprisingly. Zhang et al. (2018a) developed a one-step method to synthesize N/S co-doped graphene hydrogel, and the as-prepared hydrogel can show a capacity of 1,063 C/g at 1 A/g. Kong et al. (2018) constructed N,S-codoped graphene hydrogel with 3D hole, and the abundant in-plane pores leading to an outstanding specific capacitance of 320.0 F/g at 1 A/g.

## Concluding Remarks

In this work, the recent advances in three-dimensional graphene-based composite hydrogel were reviewed in term of their use as flexible supercapacitors electrode materials. As can be seen, much effort has been devoted in the field of 3D graphene-based composite hydrogel electrode materials. A variety of materials including metals, polymers, dopants have been reported to construct high performance graphene-based composite hydrogel, and the excellent capacitive behavior of the typical composite hydrogel can be achieved (Table 1). Nevertheless, a massive effort is still needed before real practical applications become possible. For one, the exhibited capacitance values of the hydrogel still need to be further enhanced. In particular, large scale production of graphene-based composite hydrogel with high capacitance and quality is still a challenge. More importantly, for industry-level applications, technical movements should focus on more cost-effective and straightforward approaches for the fabrication of 3D graphene-based composite hydrogel rather than the design of complicated nanomaterials. It is believed that continuous breakthroughs in the graphene-based composite hydrogel will be made with the further research and development in this exciting field, and the hydrogel can play a great role in flexible capacitive devices in the near future.

**Table 1:** Characteristics of typical graphene-based composite hydrogel for supercapacitor electrodes.

**Composite component**	**Method**	**Capacitance**	**Testing conditions**	**References**
α-MnO2 nanowire	Hydrothermal	397 F g^−1^	1.0 A g^−1^, two-electrode	Tran et al., 2017
Ni(OH)_2_ nanosheet	Hydrothermal	896 F g^−1^	0.5 A g^−1^, three-electrode	Li et al., 2019
Ni-Co hydroxide nanoneedles	Chemical reduction and hydrothermal	544 C g^−1^	2 A g^−1^, three-electrode	Hwang et al., 2018
TiO_2_ nanoparticles	Hydrothermal	372.3 F g^−1^	0.2 A g^−1^, three-electrode	Liu et al., 2017
PANI	Hydrothermal and eletrodeposition	710 F g^−1^	2 A g^−1^, three-electrode	Gao et al., 2016
PPy	Chemical reaction	363 F cm^−3^	1.0 mA cm^−3^, two-electrode	Wu and Lian, 2017
Lignosulfonate	Hydrothermal	549.5 F g^−1^	1 A g^−1^, three-electrode	Xiong et al., 2016
N dopant	Hydrothermal	387.2 F g^−1^	1 A g^−1^, three-electrode	Liao et al., 2016
N/S co-dopant	Hydrothermal	1,063 C g^−1^	1 A g^−1^, three-electrode	Zhang et al., 2018a

## Author Contributions

All authors listed have made a substantial, direct and intellectual contribution to the work, and approved it for publication.

### Conflict of Interest

The authors declare that the research was conducted in the absence of any commercial or financial relationships that could be construed as a potential conflict of interest.
